# The mix of good nutritional attitude and poor nutritional knowledge is associated with adequate intake of vitamin A and iron from wild fruits and vegetables among rural households in Acholi subregion of Northern Uganda

**DOI:** 10.1002/fsn3.800

**Published:** 2018-10-25

**Authors:** Lawrence Okidi, Walter Odongo, Duncan Ongeng

**Affiliations:** ^1^ Faculty of Agriculture and Environment Department of Food Science and Postharvest Technology Gulu University Gulu Uganda; ^2^ Faculty of Agriculture and Environment Department of Rural Development and Agribusiness Gulu University Gulu Uganda

**Keywords:** attitude, iron, Knowledge, vitamin A, wild fruits and vegetables

## Abstract

Nutritional knowledge and attitude of a population greatly affect intake of essential micronutrients. Much of the understanding on the impact of nutritional knowledge and attitude on nutrient intake has been derived from studies conducted among affluent societies using commercial food products. However, information on the link between status of nutritional knowledge and attitude on intake of essential nutrients from wild fruits and vegetables among rural communities is largely lacking. This study examined the status of nutritional knowledge and attitude associated with intake of vitamin A and iron from wild fruits and vegetables among rural households in Acholi subregion of northern Uganda. A cross‐sectional study design making use of individual household questionnaire and proximate dietary recall of a calendar year consumption period involving 248 randomly selected rural households was applied. Results showed that households had good nutritional attitude but poor nutritional knowledge. Status of knowledge was dependent on nutrition training (*p* = 0.028; coefficient, β = 0.710) and age of the respondent (*p *=* *0.044; β = −0.023), whereas status of attitude was predicted by age of the respondent (*p* = 0.014; β = 0.048). Consumption of wild fruits and vegetables contributed adequately to the dietary vitamin A and iron requirements of the households. The results have demonstrated that intake of vitamin A and iron from wild fruits and vegetables among rural households is associated with good nutritional attitude but poor nutritional knowledge. A concerted effort is necessary to improve household nutritional knowledge for better utilization of wild fruits and vegetables.

## 1 INTRODUCTION

Globally, Sub‐Saharan Africa has the highest proportion of food insecure people, with the prevalence of undernutrition standing at 23.8%. This level of undernutrition is more than twice the global undernutrition rate that stands at 11.3%. The situation is even worse for Uganda, for which the prevalence of undernutrition has been reported at 25% (FAO, IFAD, & WFP, 2014). In rural areas in Sub‐Saharan Africa, household food security is largely dependent on own production and to a lesser extent through purchase from the market (Cooper, Dimes, Rao, Shapiro, & Twomlow, 2006). However, during lean seasons or in case of crop/livestock failure, the meager food available from own production cannot support household food needs. Thus, the use of wild fruits and vegetables becomes paramount among other food insecurity coping strategies (Okori, Obua, & Baryamureeba, 2009; Pilgrim, Cullen, Smith, & Pretty, 2007). In the context of this study, wild fruits and vegetables refer to edible plants that are not managed under routine farming systems. On the other hand, in extreme circumstances such as prolonged drought or disaster situations, households rely on food donation from the government or other agencies such as the World Food Programme (Tusiime, Renard, & Smets, 2013).

Vitamin A and iron deficiencies are important nutritional constrains in many countries in Sub‐Saharan Africa (Camaschella, 2015; UNICEF, 2018; World Health Organization, 2009). In Uganda, deficiency of vitamin A and iron among vulnerable groups especially children and pregnant mothers is still very high (Uganda Bureau of Statistics (UBOS) and ICF International Inc, 2017). This suggests that households are unable to meet vitamin A and iron needs from own production and/or through food purchase. On the other hand, as is the case with several other countries in Sub‐Saharan Africa, there are areas in Uganda such as the Acholi subregion where wild fruits and vegetables exist and are routinely consumed (Acipa, Kamatenesi, & Oryem‐Origa, 2013; Oryema, 2014). Wild fruits and vegetables have been reported to be rich in micronutrients such as vitamin A and iron (Acipa et al., 2013; Oryema, 2014). Nevertheless, it is important to appreciate that information available on the utilization of wild fruits and vegetables for household nutrition among rural communities is largely qualitative (Bharucha & Pretty, 2010; Loki & Ndyomugyenyi, 2016a,b; Pardo‐de‐santayana et al., 2007). However, the actual contribution of those wild food resources in terms of the Recommended Dietary Allowance (RDA) for critical nutrients such as vitamin A and iron has largely remained unknown. Therefore, the significance of wild fruits and vegetables to nutrient needs of households that utilize them is still vaguely understood.

Empirical studies have shown that availability and access to food are essential, but they are not the only factors that ensure household food and nutrition security (Brown & Funk, 2015; Cook, Reilly, Derosa, Rohrbach, & Spruijt‐metz, 2014; Ozor, Umunnakwe, & Acheampong, 2014). Nutritional knowledge and attitude have been shown to influence dietary practices and consequently the quantity and quality of nutrients derived from a given food item (Hoogenboom, Morris, Morris, & Schaefer, 2009; Sichert‐hellert et al., 2011). Factors associated with nutritional knowledge and attitude such as choice of food, prior knowledge of expected nutritional benefits, and the frequency of consumption have been shown to influence household food and nutrition security (Carrillo, Varela, & Fiszman, 2012). Despite the well‐known significance of appropriate nutritional knowledge and attitude on food consumption behavior, it is important to appreciate that much of the understanding has been derived from studies conducted among affluent societies using commercial food products (Hoogenboom et al., 2009; Scully, Dixon, & Wakefield, 2008; Sichert‐hellert et al., 2011). However, there is very limited information on the status of nutritional knowledge and attitude associated with consumption of wild fruits and vegetables among rural households such as those in Acholi subregion of Uganda. In other words, it is largely unknown whether consumption of wild fruits and vegetables by rural households is backed by appropriate nutritional knowledge and attitude to enable the community to use wild food resources to derive critical nutrients such as vitamin A and iron. Making use of the Acholi subregion of Uganda as a study area, the objective of this study was therefore to assess the status of nutritional knowledge and attitude associated with intake of vitamin A and iron from wild fruits and vegetables among rural households.

## 2 MATERIALS AND METHODS

### 2.1 Study design and study area

**Figure 1:**
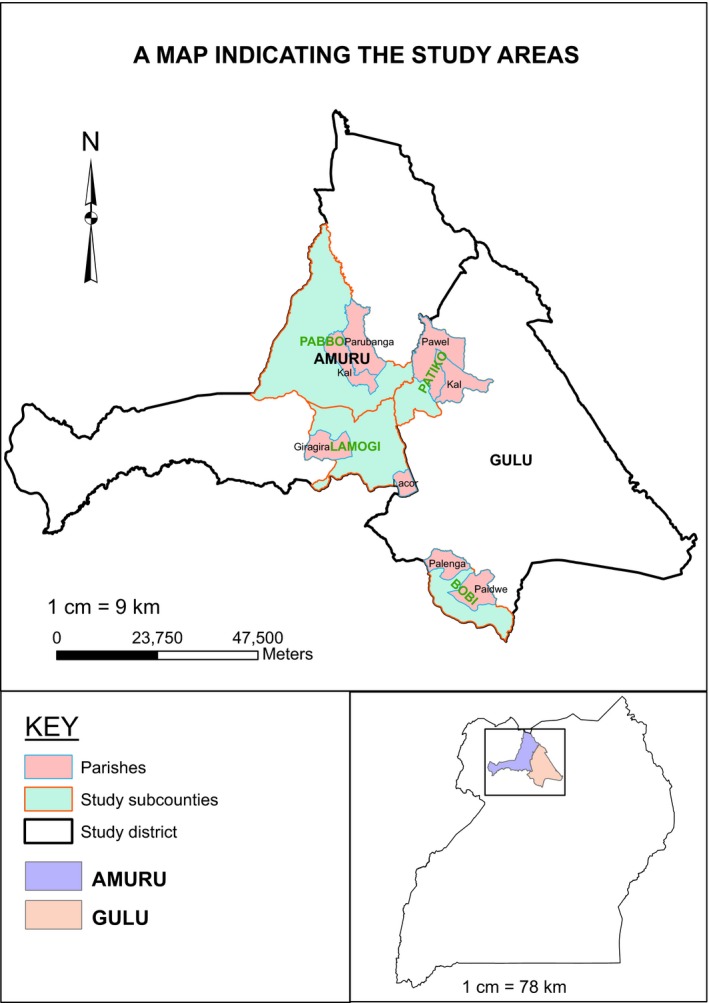
A map showing location of study area

A cross‐sectional study design employing household survey was used. The study was conducted in Amuru and Gulu districts of Acholi subregion of Uganda (Figure 1). The choice of the two districts was guided by the fact that (a) previous studies that documented consumption of wild fruits and vegetables were conducted in them and (b) communities that live in them are prone to acute food insecurity (Uganda IPC Technical Working Group, 2013). Therefore, as a coping strategy to mitigate food insecurity, communities routinely include wild fruits and vegetables in household diet (Okori et al., 2009; Oryema, Oryem‐origa, & Nanna, 2013). Gulu and Amuru districts are largely inhabited by the Acholi tribal community that speak the Acholi language. Communities in the two districts practice mainly subsistence farming as a major source of livelihood although a few large‐scale commercial farmers have inhabited farmlands of Amuru district in the recent past. Children (0–17 years) and youth (18–30 years) constitute the majority of the population (77%). The average household size is about 5.0 for both Gulu and Amuru districts. The majority of the households in Amuru (91%) and Gulu (66%) are in rural areas, and at least 51% of the population in the two districts are females (UBOS, 2016).

### 2.2 Study population and sampling framework

The study population comprised of rural households living in Amuru and Gulu districts (Figure 1). Population size of rural households inhabiting the two districts is estimated to be 472, 127 based on projection from the latest national census (UBOS, 2014). Previous statistics put the proportion of households that experienced stressed food insecurity situation in Acholi subregion at 16% (Uganda IPC Technical Working Group, 2013). On the basis of this statistic and allowing for an error of 5% (*p* = 0.05), the sample size for the study was estimated to be 207 households according to standard formula for nutritional epidemiology previously used by Kasiulevičius, Šapoka, and Filipavičiūtė (2006). Considering an attrition rate of 20%, the sample size was adjusted upwards by the same percentage and the final sample size used was 248 households. This sample size (248) is more than what had been used in similar studies conducted before (Agea, Obua, Waiswa, Okia, & Okullo, 2010; Redzic, Barudanovic, & Pilipovic, 2010).

Following determination of the sample size, a multistage sampling framework was used to select participating households. From each of the two districts (Amuru and Gulu), two subcounties were randomly selected (Gulu district: Patiko and Bobi subcounties; Amuru district: Lamogi and Pabbo subcounties), and from each of the selected subcounties, four villages were randomly selected (Patiko subcounty: Akwii, Angany, Anyadwe, and Patalira villages; Bobi subcounty: Onekdyel, Opaya, Bar, and Iraa villages; Lamogi subcounty: Opok, Ayila, Lwalakwar, and Pukure villages; and Pabbo subcounty: Centre, Oguru, Pericu, and Abera villages). Finally, 15 or 16 households were systematically selected per village to participate in the study.

### 2.3 Instrumentation and data collection

A structured, interviewer‐administered individual household questionnaire was used. The questionnaire consisted of four sections: the first section assessed socio‐demographic characteristics of the households; the second section assessed nutritional knowledge using eight statements anchored on a 3‐point Likert scale; the third section assessed attitude toward wild fruits and vegetables using 13 statements anchored on a 3‐point Likert scale; and the fourth section collected data on wild fruits and vegetables (amounts harvested, sold, given away, consumed, frequency with which they were consumed, duration over which they were consumed and overall consumption in an agricultural year). The questionnaire also had provision for information on composition of household members by age, physiological status (for pregnant and lactating mothers), and gender. The constructs used to measure nutritional knowledge and attitude were adapted with modification from previous studies (Bukusuba, Kikafunda, & Whitehead, 2010; Damodaran, Parkin, & Fennema, 2008; Pilgrim et al., 2007). Literature study and laboratory analyses were undertaken to collect information on nutrient contents of the wild fruits and vegetables routinely consumed in the community. Before conducting laboratory analyses, thorough review of peer‐reviewed papers for existing data on vitamin A/beta‐carotene and iron was carried out to avoid duplication (Acipa et al., 2013; Howard, Talcott, Brenes, & Villalon, 2000; Okullu et al., 2010; Oryema, 2014; Otunola, Oloyede, Oladiji, & Afolayan, 2010; Srivastava, 2011). In situations where information was missing, laboratory analyses for the aforementioned nutrients were conducted according to AOAC (1990) and Rodriguez‐Amaya and Kimura (2004).

Before the actual data collection, the questionnaire was pretested and adjustments made before the final version could be produced. Questions that were difficult to interpret by the respondents and did not provide valid results were adjusted accordingly. The study made use of interviewers that had undergone prior training in individual household method (Petty & Ellis, 2015) and had experience in nutrition survey. To ensure proper data collection on consumption, the interviewers were trained on questionnaire administration and on techniques of translating local measurements of assessing food quantity to standard measures (grams). Because not all parts of fruits and vegetables are consumed, the interviewers were also trained on how to adjust the quantity measured for edible portion. Actual data collection took place between November and December 2015. The main respondents were women. This is because in a rural setting as is the case in the study area, women are responsible for collection and preparation of food in households (Hyder et al., 2005). In situations where women were unavailable (e.g., the widowed, divorced, the woman being absent for some other reasons), men were interviewed.

### 2.4 Data analysis

#### 2.4.1 Status of nutritional attitude and knowledge

Data were coded and analyzed using Statistical Package for Social Scientist (SPSS) version 20.0. Before actual analysis, negatively worded questions were reverse‐coded. Descriptive statistics (frequencies and percentages) were used to analyze socio‐demographic characteristics. Knowledge was scored on a 2‐point scale (0 score for “wrong” or “don't know” response and 1 for correct response). Knowledge scores were then expressed as a percentage of total score (8). Attitude was scored on a 3‐point scale (disagree, not sure, and agree). The scores ranged from zero for the most negative (disagree) to 2 for the most positive (agree). Attitude scores were expressed as a percentage of the total points‐26 (Masuku & Lan, 2014). Scores below 50% and ≤ 57% were considered as poor for knowledge (Bas, Ersun, & Kıvanc, 2006) and attitude (Ul Haq, Hassali, Shafie, Saleem, & Farooqui, 2012), respectively. Otherwise above the stated cut‐offs, those parameters were considered to be good. Bivariate analysis (Pearson's correlation) was used to determine the association between knowledge and attitude. Binary logistic regression was then used to determine socio‐demographic predictors of good nutritional knowledge and attitude. The level of statistical significance was fixed at 5% (*p ≤ *0.05).

#### 2.4.2 Contribution of wild fruits and vegetables to vitamin A and iron requirements

To determine the contribution of wild fruits and vegetables to household vitamin A and iron requirements, Recommended Dietary Allowance (RDA) for vitamin A and iron for all the members (of different age groups, physiological status, and sex) in a given household was computed for a day (Brown et al., 2011), constituting the pooled household RDA. The pooled daily requirement for each household was further standardized to pooled annual requirement (365 days). The quantity of each wild fruit and vegetable consumed per day based on local measurement was converted to the standard measure (grams), adjusted for edible portion, and computed for the duration over which it was consumed thus leading to annual quantity consumed. Using literature or laboratory values (Table 1), the quantity of vitamin A and iron consumed from each fruit or vegetable was calculated from the quantity of each item consumed annually and summed up for each household. The contribution of wild fruits and vegetables to annual household dietary requirement was then computed as a proportion of the household RDA for each nutrient. A key assumption in this analytical framework is that food distribution in a household reflects individual food needs.

**Table 1:** Vitamin A and iron content of wild fruits and vegetables used in the study

Local name	Scientific name	Vitamin A (μg/100 g)	Iron (mg/100 g)	References
Wild fruits				
Oywello	*Vitex doniana* Sweet	60	0.33	Oryema (2014)
Yaa	*Vitellaria paradoxa C.F. Gaertn*	228.3	0.86	Oryema (2014)
Tugu	*Borassus aethiopum Mart*	445.8	0.62	Oryema (2014)
Oceyo	*Aframomum angustifolium (Sonn.) K.Schum*.	2236	6660	Acipa et al. (2013)
Cwaa	*Tamarindus indica L*.	1391	45180	Acipa et al. (2013)
Kalara	*Capsicum frutescens L*.	98.92	3.39	Otunola et al. (2010); Howard et al. (2000)
Tongogwal Madito	*Physalis macrantha Link*	172.78	29.05	Determined in this study
Kano	*Syzygium malaccense (L.) Merr. & L.M.Pe*	207.81	35.54	Determined in this study
Wild vegetables				
Gwanya	*Hibiscus acetosella*	178.12	55.78	Determined in this study
Ayuyu	*Acalypha bipartita*	16790	21960	Acipa et al. (2013)
Oyado	*Senna obtusifolia*	253.16	234.6	Determined in this study
Otigo lum/nyim	*Corchorus trilocularis*	361.11	85.51	Determined in this study
Obuga lum	*Amaranthus spinosus*	58600	13.28	Srivastava (2011)
Pot kalara	*Capsicum frutescens L*.	2118.25	29.19	Determined in this study
Malakwang Odwonga	NA	1044.80	56.66	Determined in this study
Layika	*Corchorus olitorius*	1768.00	76.05	Determined in this study
Boo ayom/ lok	NA	973.71	70.56	Determined in this study

*Note*

NA, Scientific name is not available.

## 3 RESULTS

### 3.1 Socio‐demographic characteristics

Data on socio‐demographic characteristics of the respondents that participated in the study are presented in Table 2. Majority (72%) of the respondents were women and nearly half of the total respondents were youth. In terms of marital status, over 80% of the respondents were married and the rest fell in the category of widowed, single, and divorced in decreasing order of magnitude. From a religious perspective, about 81% of the respondents were of Catholic denomination and the rest were Pentecostals, Protestants, and Muslims in decreasing order of magnitude. More than ¾ of the respondents were involved in farming as the main occupation and the remaining fraction consisted of civil servants, casual laborers, traders, and retired pensioners in decreasing order of magnitude. Most of the respondent (63%) had attended only primary level of education while nearly 30% were graduates of secondary and tertiary education and the remaining fraction never attended any formal education at all. Over 60% of the respondents had never attended nutrition education, while more than half (64%) of them had interacted with the village health team (VHT).

**Table 2:** Socio‐demographic characteristics of the study participants

Variable	%	Variable	%
Gender		Religion	
Male	28.1	Catholic	80.7
Female	71.9	Protestant	7.3
Age		Muslim	2.6
≤18	3.1	Pentecostal	9.4
19–35	48.4	Occupation	
36–45	18.2	Farmer	78.7
≥46	30.2	Casual labourer	6.7
Marital status		Retired with pension	1.3
Married	82.3	Trader	5.3
Single	3.6	Civil servant	8.0
Widowed	11.5	Education level	
Divorced	2.6	No formal education	7.8
Distance to the nearest trading centre (Km)		Primary	62.5
≤2.9	48.4	O Level	16.1
3.0–5.9	39.6	A Level	5.2
≥6	12.0	Tertiary/ University	8.4
Nutrition education		Interaction with VHT	
Never	62.5	Yes	63.5
1 time	23.4		
≥2 times	14.1		

*Note*

VHT, village health team.

### 3.2 The status of nutritional knowledge and attitude

The mean score for nutritional knowledge and attitude was 48% (3.84) and 60% (15.63), respectively (Table 3). The level of nutritional attitude was good, whereas knowledge was bad on the basis of cut‐offs presented under subsection 2.4.1.

**Table 3:** The level of nutritional knowledge and attitude

Variable	Total score	Minimum score	Maximum score	Mean	*SD*
Knowledge	8	1	7	3.84	1.18
Attitude	26	4	22	15.63	1.88

Table 4 presents data on the distribution of responses to questions testing nutrition knowledge. Regarding nutritional values, more than 80% of the respondents were aware that wild fruits and vegetables are a good source of macronutrients, while nearly 71% perceived wild fruits and vegetables to have medicinal values. However, nearly half of the respondents did not believe that wild fruits and vegetables should be consumed on a daily basis.

**Table 4:** Distribution of responses to questions testing specific aspects of nutritional knowledge

Variable	Agree %	Don't know %	Disagree %
WFVs are a good source of energy	2.6	10.9	86.6
WFVs have medicinal values	71.4	22.9	5.7
WFVs protects me against diseases (sickness)	71.9	16.7	11.5
Nutrients cannot be provided by only one type of food	60.4	5.7	33.9
Stems of vegetables are rich in nutrients	33.9	31.8	34.4
Some WFVs have anti‐nutritional factors^a^	44.3	26.0	29.7
It is important to consume fruits and vegetables on a daily basis	41.7	7.8	50.5
Wild fruits and vegetables are a good source of protein	74.5	17.2	8.3

*Notes*

WFVs, wild fruits and vegetables.

a Item was reverse‐scored.

Data on the distribution of responses to questions testing nutritional attitude are presented in Table 5. Majority of the respondents (82%) considered wild fruits and vegetables to be safe for consumption notwithstanding the fact that the same proportion considered wild fruits and vegetables not to be nutritious. Nevertheless, only less than 10% of the respondents did not like the taste of wild fruits and vegetables. About 72% of the respondents considered wild fruits and vegetables not to be expensive although 30% of the respondents anticipated consumption of wild fruits and vegetables to be unsustainable. Interestingly, a greater proportion of the respondents (80%) was ready to consume wild fruits and vegetables as supplements for domestically produced foods. On the other hand, 56% of the respondents were willing to consume wild fruits and vegetables as a substitute for those that are domestically cultivated. Last but not least, there was no association between nutritional knowledge and attitude (*p *=* *0.115).

**Table 5:** Distribution of responses to questions testing specific aspects of nutritional attitude

Variable	Disagree %	Not sure %	Agree %
I anticipate wild fruits and vegetables to be unsafe^a^	8.9	9.4	81.8
I anticipate wild fruits and vegetables to have bad taste^a^	2.6	2.6	94.8
I anticipate wild fruits and vegetables not to be nutritious^a^	8.9	9.4	81.8
I anticipate wild fruits and vegetables to be expensive^a^	26.6	1.6	71.9
I anticipate the consumption of WFVs to be unsustainable^a^	29.7	9.4	60.9
I anticipate wild fruits and vegetables to be for the needy^a^	26.6	1.6	71.9
I anticipate wild fruits and vegetables to be fake food^a^	19.8	3.6	76.6
I would eat wild fruits and vegetables as a supplement for domestically produced foods	15.6	4.2	80.2
I would eat wild fruits and vegetables during periods of good harvest of domestically produced foods	27.6	8.9	63.5
I would eat wild fruits and vegetables as a substitute for domestically produced food	34.9	9.4	55.7
Vegetables must be overcooked to kill microbes^a^	75.5	6.3	18.2
I should only eat fruits and vegetables when I feel like^a^	70.3	6.3	23.4
I can obtain all the nutritious food from my own garden^a^	25.5	11.5	63.0

a Item was reverse‐scored.

### 3.3 Socio‐demographic predictors of nutritional knowledge and attitude

The results of binary logistic regression analysis executed to determine socio‐demographic predictors of nutritional knowledge and attitude are presented in Table 6. Nutritional knowledge was determined by attendance of nutritional training (*p* = 0.028) and age of the respondent (*p* = 0.044). Attendance of nutrition training increased nutritional knowledge by 71% while an increase in age by 1 year decreased nutritional knowledge of the respondent by 2%. Nutritional attitude was only influenced by age of the respondent (*p* = 0.014). An increase in age by one year improved the status of nutritional attitude by 5%.

**Table 6:** Relationship between good knowledge and attitude and Socio‐demographic variables

Independent variable	Knowledge		Attitude	
	Coefficient (B)	*p*‐value	Coefficient (B)	*p*‐value
Gender	−0.619	0.083	−0.417	0.481
Nutrition training	0.710	0.028^a^	0.643	0.241
Age	−0.023	0.044^a^	0.048	0.014^a^
Distance to nearest market	−0.029	0.416	−0.009	0.917
Household size	0.036	0.496	0.611	0.314
Group membership	−0.161	0.663	−0.362	0.529
Interaction with VHT	−0.123	0.724	−0.150	0.302

*Notes*

VHT, village health team.

a Values are significant at 5%.

### 3.4 Contribution of wild fruits and vegetables to household intake of vitamin A and iron

**Figure 2:**
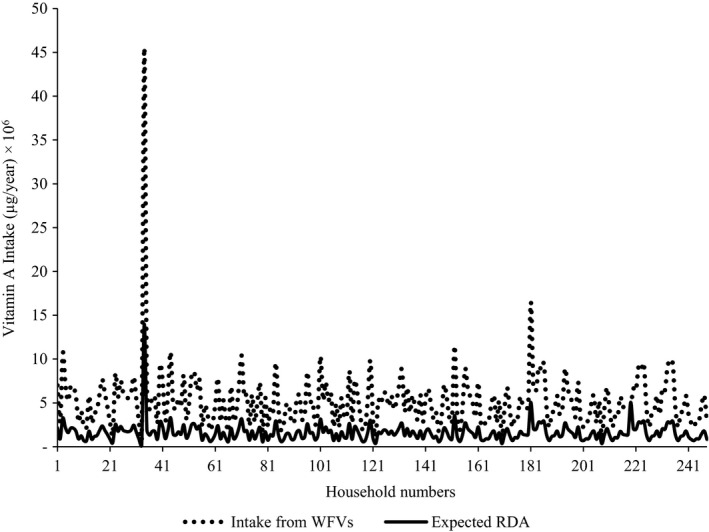
Contribution of wild fruits and vegetables to household dietary vitamin A requirement

**Figure 3:**
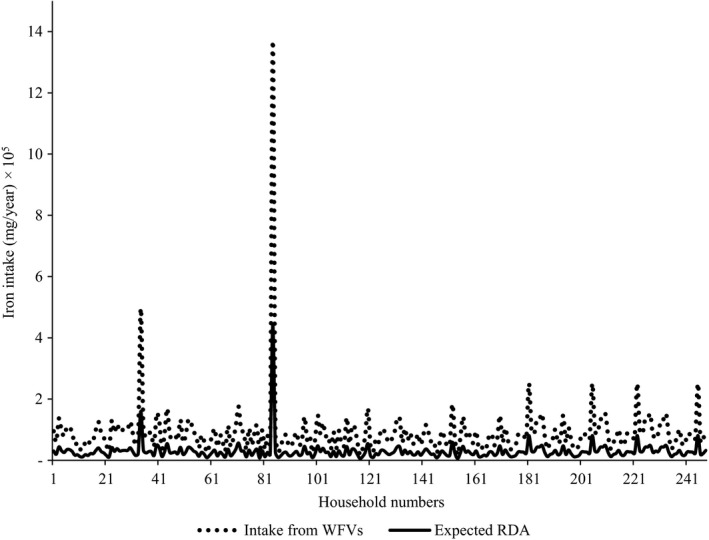
Contribution of wild fruits and vegetables to household dietary iron requirement

The contribution of wild fruits and vegetables to the pooled household requirement for vitamin A and iron is presented in Figures 2 and 3, respectively. In the case of vitamin A, all the households met the RDA while about 70% of them derived the micronutrient more than the RDA from wild fruits and vegetables. Similarly, as was the case for vitamin A, most of the households achieved sufficient dietary intake of iron from wild fruits and vegetables. In fact, more than half of the households derived more than twice the RDA for iron from those wild food resources.

## 4 DISCUSSION

This paper examined the status of nutritional attitude and knowledge associated with vitamin A and iron intake from wild fruits and vegetables in a rural setting where wild food resources are important for food security. We particularly assessed whether vitamin A and iron intake from wild fruits and vegetables in the community was backed up by appropriate nutritional knowledge and attitude to enable households derive better nutritional benefits from them. This study revealed that nutritional knowledge associated with utilization of wild fruits and vegetables among households was generally poor. This implies that rural households in Acholi subregion of Uganda have inadequate understanding of the nutritional benefits that can be derived from wild fruits and vegetables. A sizable number of households (71%) had basic knowledge of the fact that fruits and vegetable protect humans from diseases and the need to consume a wide diversity of food groups. However, there was generally poor knowledge about the role of fruits and vegetables as a major source of micronutrients and the need to consume fruits and vegetables on a daily basis in order to derive better nutritional benefit. The poor status of knowledge among households on nutritional importance of fruits and vegetables and of wild fruits and vegetables in particular observed in this study could be explained by the changing life patterns that disregard wild food resources thereby hindering their utilization (Oryema, Oryem‐origa, & Roos, 2015). The low level of nutritional knowledge regarding wild fruits and vegetables could also be explained by lack of information on nutritional characteristics of wild food resources available for use by the community in the study area. A concerted effort is therefore necessary to foster comprehensive documentation of nutritional characteristics of wild food resources. Such information would be of great use in community nutrition education.

The poor level of nutrition knowledge observed in this study is in contrast with findings from other nutrition studies. For instance, Anand and Puri (2013) and Das and Mukherjee (2014) found that women's nutritional knowledge on HIV management and infant feeding practices was good among urban and rural Indian population, respectively. This implies that nutritional knowledge varies with context under which it is being applied. Attendance of nutrition training and age were critical determinants of household nutritional knowledge. The negative influence of age on the level of nutritional knowledge observed in this study could be explained by the dimension of knowledge studied. Whereas knowledge about the diversity of wild fruits and vegetables has been suggested to increase with age (Ghosh‐Jerath, Singh, Kamboj, Goldberg, & Magsumbol, 2015; Pilgrim et al., 2007), knowledge about nutritional values of fruits and vegetables seems more comprehended by the younger than the older generation. This assertion finds credence from data which show that close to 90% of the respondents had attended formal education. Such respondents could have acquired basic knowledge on nutrition from science or biology which is a compulsory subject in primary and ordinary secondary level of education, respectively, under the current Ugandan education system.

It was interesting to note that attitude of the respondents toward consumption of wild fruits and vegetables was good. This observation could be explained by the fact that wild fruits and vegetables contribute significantly to household food security in the study area (Loki & Ndyomugyenyi, 2016b; Oryema et al., 2013). In addition, availability of those wild food resources within the vicinity of the study participants (rural areas) could also explain households’ good attitude toward them (Msuya, Kideghesho, & Mosha, 2010; Pilgrim et al., 2007). This observation contrasts sharply with findings from other studies conducted in Spain and Ethiopia where consumer attitude toward consumption of wild fruits and vegetables was poor (Giday, Asfaw, Elmqvist, & Woldu, 2003; Pardo‐de‐santayana, Tardio, & Morales, 2005). This disparity illustrates the differences in the nature of attitude that exist among communities between different geographic locations. By implication therefore, it becomes apparent with regard to wild fruits and vegetables that information on attitude obtained from a given geographic location may not be applicable in another location, thus providing further justification for the current study. Culture is an important factor that modulates consumer attitude toward a particular food type (Kruger & Gericke, 2002; Thurber et al., 2016). It is therefore plausible that cultural differences between communities in Acholi subregion of Uganda and those in Ethiopia and Spain where the respective studies were conducted could explain the disparity. A peculiar observation from this study is that nutritional attitude was only determined by age of the respondent and in a positive way. Improvement in household nutritional attitude with age could be explained by the mere‐exposure effect theory (Jones & Kervin, 2010; Kumar, Onufrak, Zytnick, Kingsley, & Park, 2014; Scully et al., 2008). This is a psychological phenomenon by which people tend to develop a preference for things merely because they are familiar with them. In the context of this study, the more the number of years the respondent used wild fruits and vegetables, the more he/she got used to wild food resources and hence the better the attitude toward them.

The fact that adequate nutrient intake was associated with good nutritional attitude but poor knowledge challenges the common belief that good knowledge manifests into good attitude (Anand and Puri (2013). The observed results could be a consequence of limited nutrition education received by the households. This is evidenced by the fact that up to 63% of the households never attended any nutrition education while only 24 and 14% received nutrition education once and twice, respectively. Multiple attendance of nutrition education has been shown to improve nutrition knowledge (Eyles & Mhurchu, 2011; Pomerleau, Lock, Knai, & McKee, 2005). Lack of correlation between nutritional knowledge and attitude revealed by the current study deviates from the findings of other nutrition‐related studies conducted before. For instance, Masuku and Lan (2014) observed a significant correlation between nutritional knowledge and attitude in a study conducted to assess the integration of nutrition in management of HIV in Swaziland. However, the results of Masuku and Lan (2014) cannot be compared directly with the finding from the current study due to differences in context between the two studies.

Vitamin A supplied by wild fruits and vegetables was generally above the annual RDA of households. This finding concurs with the results of the work conducted by Redzic (2006) a decade ago in Bosnia‐Herzegovina. Nevertheless, there are also other contrasting findings published by other authors. For instance, Oiye, Shiundu, and Oniang'o (2009) showed that households in rural areas of western Kenya obtained only up to 65.7% of vitamin A from African leafy vegetables. In addition, work conducted by Bélanger, Balakrishna, Latha, Katumalla, and Johns (2010) revealed that rural households in India were able to meet only 40% of RDA for vitamin A from uncultivated vegetables. Despite differences, comparing results of the current study with others cited is not appropriate because of the differences in types of fruits and vegetables encountered in each study. Similarly, iron intake from wild fruits and vegetables studied was also above the annual household RDA and compares favorably well with data previously published by Srivastava (2011) regarding rural inhabitants in India. The fact that rural households in Acholi subregion derived vitamin A and iron at levels above the RDA is not surprising. This finding is adequately backed by the outcome of the Uganda demographic health survey which showed that vitamin A and iron were not among the nutrients of public health importance in the subregion (Uganda Bureau of Statistics (UBOS) and ICF International Inc, 2012).

Despite the significance of wild fruits and vegetables in supplying critical nutrients such as vitamin A and iron to rural households as illustrated in this study, poor natural resource management in many countries in Sub‐Saharan Africa is still a serious bottleneck. Lack of sustainable livelihood strategies has forced rural communities to destroy natural habitats in which wild fruits and vegetables proliferate. Typical example is indiscriminate tree‐cutting for charcoal production (Akena, 2012). From economic point of view, national governments do spend a lot of resources in ameliorating vitamin A and iron deficiency through medical supplementation in areas where such micronutrients are endemically deficient. Therefore, strategic interventions are required to protect natural ecosystems within communities that are endowed with wild fruits and vegetables. Otherwise, future sustainability of wild food resources in supplying rural communities with critical nutrients such as vitamin A and iron will be compromised.

There are three limitations inherent in this study. First, substantial information on the content of vitamin A and iron for wild food resources studied was derived from literature. Considering the fact that nutritional composition of fruits and vegetables depends on geographic location (Okello, 2010; Okullu et al., 2010) and variety (Howard et al., 2000), future studies should consider using data from real‐time laboratory analysis. Secondly, it is known that fruits and vegetables generally contain antinutritional factors that lower bioavailability of nutrients, and in the context of this study, iron in particular (Rathod & Valvi, 2011; Umaru, Adamu, Dahiru, & Nadro, 2007). This implies that the contribution levels reported in this study are to some extent theoretical. Future studies should consider contribution based on iron which is biologically available. Thirdly, the assumption that food distribution in households is in accordance with individual household member food needs may not hold. Thus, nutrient adequacy determined based on pooled household estimate may not reflect intrahoused nutrient adequacy.

## 5 CONCLUSION

This study has demonstrated that intake of vitamin A and iron from wild fruits and vegetables among rural households is associated with good nutritional attitude but poor nutritional knowledge. Despite the mix of good nutritional attitude and poor nutritional knowledge, and taking into account the limitations of the study, theoretically, households derived more than adequate amount of vitamin A and iron from the wild fruits and vegetables studied. Attitude but not knowledge seems to be a key impetus that enabled rural households to meet vitamin A and iron requirements through consumption of wild fruits and vegetables.

## CONFLICT OF INTEREST

The authors declare no conflict of interest.

## ETHICAL STATEMENTS

**Ethical review:** Ethical clearance was obtained from Gulu University Research Ethics Committee (GUREC/04/09/16). Authorization to conduct the study in each of the two districts was obtained from the Chief Administrative Officer of the respective districts.

**Informed consent:** Participation in the study was voluntary. Full consent of the participants was sought before the interview, and each participant signed a consent form before participating in the study.
